# From principles to practice: Clarifying new obligations under Victoria’s *Mental Health and Wellbeing Act 2022*

**DOI:** 10.1177/10398562241251595

**Published:** 2024-04-30

**Authors:** Simon Katterl

**Affiliations:** Simon Katterl Consulting, Naarm (Melbourne), VIC, Australia

**Keywords:** human rights, clinical governance, leadership, mental health law

## Abstract

**Objective:**

To explain the new test for complying with the mental health principles under the *Mental Health and Wellbeing Act 2022* (Vic).

**Conclusion:**

The principles carry over limitations from the previous *Mental Health Act 2014* (Vic) while also containing new features. The ‘all reasonable efforts to comply’ and ‘proper consideration’ tests resemble the existing test under section 38(1) of the *Charter of Human Rights and Responsibilities Act 2006* (Vic) that also apply to public mental health services. Taking these duties together, public mental health services, including hospital and community mental health boards, clinical directors and clinical governance processes, will need to show concrete evidence of specific rights and/or principles being deliberated in their decisions.

On 1 September 2023, the *Mental Health and Wellbeing Act 2022* (Vic) (**the Act**) took effect, replacing the *Mental Health Act 2014* (Vic) (**the previous Act**). After mental health services had not complied with the previous Act,^1^ the latest legislative reform was met with a more muted response from experts^2^ and concern that mental health clinicians had not been adequately prepared to understand their new duties.^3^ One of the novel elements of the Act requiring explanation is the introduction of new mental health principles. These principles are similar to co-existing duties under the *Charter of Human Rights and Responsibilities Act 2006* (Vic) (**the Charter**), which have also not been complied with by the Victorian Government and public mental health services.^4^ These changes carry learning opportunities for other jurisdictions who hold both human rights and mental health laws^5^ and who consider using principles in their mental health law reforms.^6^

This paper provides an overview of the new principles in the Act and how Charter obligations require a functional positive duty to demonstrate how rights are complied with, not just in clinical decision-making, but also in the broader governance of a health service. The paper begins with a brief account of the failures under the previous Act and the Charter. Next, it highlights introducing new mental health principles into the Act and the duties and enforcement mechanisms available. Finally, it closes by providing practical advice about how practitioners and operational leaders can demonstrate compliance with these duties. Such advice may inform how human rights are implemented at service-levels in various Australian and New Zealand contexts.

## Where we have come from

The Royal Commission into Victoria’s Mental Health System held that the mental health system had ‘catastrophically’ failed to meet expectations.^7^ Among the many issues were discriminatory mental health laws and a failure by services to comply with the minimum standards within those laws.^1^ Oversight bodies such as the Mental Health Complaints Commissioner (**previous Commission**) have been criticised for failing to perform their role to protect human rights.^8^

The system had also failed to adhere to its human rights obligations under the Charter. The Charter is one of three human rights instruments passed in the Australian Capital Territory, Queensland and Victoria, with the latter’s legislation containing 20 human rights (detailed in Table 1).^9^ Section 38(1) of the Charter, which regulates public authorities, including the Victorian Department of Health and Victorian public mental health services, states that ‘it is unlawful for a public authority to act in a way that is incompatible with a human right or, in making a decision, to fail to give proper consideration to a relevant human right’. In effect, this provision has two duties. First, public mental health services must ensure any limitations on rights are justifiable with reference to specific steps set out in section 7(2). Second, public mental health services must be able to show that where their decisions engage a relevant human right, they must be able to produce evidence that they had indeed given adequate consideration to specific human rights. This duty does not just apply at the point of clinical decision-making but also at the point of public hospital and community mental health board, clinical governance, service design, training and other levels that can have a material impact on human rights at the point of service delivery. The duty requires decision-makers to think about how their decisions today, will have human rights impacts tomorrow.

**Table 1.** *Rights under the Charter of Human Rights and Responsibilities Act 2006* (Vic)

• Recognition and equality before the law
• Right to life
• Protection from torture and cruel, inhuman or degrading treatment
• Freedom from forced work
• Freedom of movement
• Privacy and reputation
• Freedom of thought, conscience, religion and belief
• Freedom of expression
• Peaceful assembly and freedom of association
• Protection of families and children
• Taking part in public life
• Cultural rights
• Property rights
• Liberty and security of person
• Humane treatment when deprived of liberty
• Children in the criminal process
• Fair hearing
• Rights in criminal proceedings
• Right not to be tried or punished more than once

The Victorian Government and public mental health services have often failed to comply with this duty.^4^ The Victorian Government had not shown proper consideration of relevant human rights across various parts of its systems management and service commissioning processes. Public mental health services’ models of care, how they are operationalised in risk-averse environments, and how clinical governance processes monitor them often fail to show proper consideration of relevant Charter rights.^4^ These failures in public administration and clinical governance need change as part of a new mental health system.

## The introduction of the Act

The passing of the Act presaged the creation of a range of new bodies as well as changes to the previous Commission, which now has a broader focus on stigma reduction and systems monitoring, with powers to conduct own-motion inquiries under the new name of the Mental Health and Wellbeing Commission (**new Commission**). The Act also included changes to mental health consumers’ rights, though they are minor.^2^

Changes to the mental health principles were one such rights-related change. Under the previous Act, decision-makers exercising a function under the Act had to ‘have regard’ to the principles. The new Act and its principles create a longer list of 13 mental health principles (detailed in Table 1) and treatment decision-making principles to apply to treatment decision-making (not covered in this article).

The new test under the Act is that mental health and wellbeing service providers, ‘exercising a function under the Act’ and ‘making a decision under this Act’, must ‘make all reasonable efforts to comply’ with the principles and give ‘proper consideration’ to the principles. The explanatory notes when the Act was being debated in Parliament outlined the intention behind this new test is ‘aligning with the test in the Charter’, which has 20 human rights detailed in Table 2. In some respects, such as the explicit statement that it cannot give rise to a cause of action in court for breach (whereas the Charter can if attached to a breach elsewhere in other legislation) and that it is ‘make all reasonable efforts to comply’ rather than ‘comply’ (the test in the Charter), suggest this is not wholly true. Nevertheless, the test for the principles in the Act is similar enough to the Charter that it is a worthy topic of exploration.

**Table 2.** Mental health principles under the *Mental Health and Wellbeing Act 2022* (Vic)

• Dignity and autonomy principle
• Diversity of care principle
• Least restrictive principle
• Supported decision-making principle
• Families and carers principle
• Lived experience principle
• Health needs principle
• Dignity of risk principle
• Wellbeing of young people principle
• Diversity principle
• Gender safety principle
• Cultural safety principle
• Wellbeing of dependents principle

There are important enforcement and monitoring processes attached to these obligations. The new Commission will, like the previous Commission, have the power to handle complaints under the Act. This remit includes complaints about compliance with other provisions of the Act, about the provision or non-provision of services, about compliance with these principles and about the failure of a mental health service to deal with an internal complaint properly. Mental health services will also need to demonstrate how they have sought to give effect to one or more principles under the Act in their annual report.

The value of the principles and the Charter obligations is that they impose a duty on mental health service providers to seek to proactively address possible human rights issues. They do so by generating the duty at all levels of service delivery – with the requirement to demonstrate it with evidence. The challenge that this poses is that these legal tests can be, at times, cumbersome. Therefore, guidance on how and where hospital and community mental health boards and executives can practically comply with obligations under the Charter and the Act’s principles is crucial.

## Translating the law into practice

Mental health and human rights laws are often criticised for being cumbersome and confusing. This may partly explain why the introduction of mental health principles has failed to change practice previously.^5^ A new model – called the Human Rights at the Heart Model (**HRatH Model**) has been developed^10^ to translate *legal obligations* into *behaviours* that leaders can engage in to more easily comply.

The HRatH model has three core steps designated ‘Forecast’, ‘Assess’ and ‘Decide’, which are defined in Table 3.^10^ It’s crucial that personnel working at the executive and governance level apply this step wherever rights may be engaged (which may be more common than currently understood).

**Table 3.** Steps to comply with the charter and principles

	Question to ask at each step
Forecast the impacts of today’s decision	• How could people at the end of our decision or policy be impacted?
	• How can we draw on lived experience in this decision?
Assess the human rights situation	• What is the human rights context and history regarding this decision, policy or area?
	• Are any rights limited or lacking?
Decide on how to proceed	• How do we promote, comply and balance human rights?
	• How will we keep records to show that we considered human rights?

There are many ways that this duty applies to ongoing governance, design and operations of public mental health services.^9^ For example, various rights and principles require decision-makers to forecast how training, quality and safety processes, models of care, ward processes, complaints procedures and more already limit human rights associated with coercion and consider what steps are necessary to address this. An absence of policies, training or daily reminders to staff might also mean that people are excluded from care or provided a lower standard of care based on their gender, mental health diagnosis, drug or alcohol use or disability. Addressing workforce safety issues is a common area of concern for managers, with the law requiring that they ensure any safety measures comply with the obligations to properly protect the rights of people detained within services. Further examples are provided in Table 4.

**Table 4.** Examples of where human rights duties apply at governance, executive and management levels

Level where this may arise inside a service	Examples of possible rights-impacting decisions
Hospital or community mental health board decisions	• Decisions about prioritising human rights in strategic planning
	• Setting goals on the elimination of coercion from services
	• Establishing internal reporting mechanisms from the CEO on human rights measures
Executive and leadership	• Attaching specific rights-related responsibilities to all or specific staff members and/or managers
	• Communications from executives and leadership on the importance of human rights
Service design	• New services are designed to eliminate existing rights limitations in current mental health services
	• New services are designed so that barriers to access and equality – such as those for Indigenous people, culturally and linguistically diverse people, trans and gender diverse people and more – are addressed
	• Development of models of care, policies and protocols to support human rights
Clinical governance and risk management	• Clinical governance processes routinely monitor the compliance of mental health practitioners with their obligations under the Charter and the Act
	• Clinical risk management processes and whether any limitations on rights in them, or balancing of different peoples’ rights, are legally justifiable
Staff recruitment, training and performance management	• The selection of staff and whether knowledge and competencies around human rights competencies are required
	• The training and professional development for staff, including monitoring whether existing training in other areas (such as risk assessment and management processes) are compliant with human rights
	• Performance review and management processes for staff where they are shown to have resistance to human rights requirements in their role

The HRatH three-step model (Forecast, Assess and Decide) may offer a helpful framework and serves as a constant reminder. It not only helps organisations adhere to the law and enhance governance and operational decisions but also underscores the fact that legal obligations and the risk of non-compliance extend beyond clinicians. Board members and executive staff who establish working conditions for mental health workers also share in the responsibility. Those board and executive staff should comprise of, and collaborate with, consumer workforce members.

## Conclusion

The new mental health laws in Victoria build upon existing duties outlined in the Charter. The scale of legislative change in these reforms should not be overstated and reformers should learn from the failure of the previous Act that rights and principles are not self-executing. Their achievement also requires meaningful evaluation based on periodic reviews and strong alignment with international human rights measures^5^ and improved regulatory oversight.^8^ Nevertheless, renewal of mental health laws and principles provide an opening to challenge the status quo in mental health services by emphasising the importance of leadership in preventing human rights issues before they manifest. The HRatH model may provide a way for leaders to practically bring a human rights focus to their daily decision-making.
